# Molecular Link between DNA Damage Response and Microtubule Dynamics

**DOI:** 10.3390/ijms23136986

**Published:** 2022-06-23

**Authors:** Jung Min Kim

**Affiliations:** Department of Pharmacology, Chonnam National University Medical School, Gwangju 58128, Korea; jungminkim@jnu.ac.kr

**Keywords:** microtubules, DNA damage response, centrosome, nuclear reorganization, chromatin mobility, DNA repair

## Abstract

Microtubules are major components of the cytoskeleton that play important roles in cellular processes such as intracellular transport and cell division. In recent years, it has become evident that microtubule networks play a role in genome maintenance during interphase. In this review, we highlight recent advances in understanding the role of microtubule dynamics in DNA damage response and repair. We first describe how DNA damage checkpoints regulate microtubule organization and stability. We then highlight how microtubule networks are involved in the nuclear remodeling following DNA damage, which leads to changes in chromosome organization. Lastly, we discuss how microtubule dynamics participate in the mobility of damaged DNA and promote consequent DNA repair. Together, the literature indicates the importance of microtubule dynamics in genome organization and stability during interphase.

## 1. Introduction

Microtubules that form a part of the cytoskeleton are involved in multiple cellular processes, including cell division, intracellular transport, cell motility, and cell shape [1,2]. Microtubules are formed from protein subunits of tubulin, and each tubulin protein consists of two subunits, α-tubulin and β-tubulin. Microtubules are highly dynamic structures that rapidly oscillate between phases of polymerization and depolymerization by the addition or removal of tubulin proteins [2]. Microtubules are nucleated from tubulin subunits at specific subcellular locations, mainly the centrosomes or MTOCs (microtubule organizing centers) [1,3]. Upon nucleation, there are many proteins that bind to microtubules, including the motor proteins dynein and kinesin, and other proteins important for regulating microtubule dynamics [4]. The microtubule cytoskeleton has a central role in cell division by forming the mitotic spindles that segregate chromosomes [5,6]. Besides its function during mitosis, recent publications have shown that microtubules also affect chromosome structure during interphase and play a role in genome maintenance [7,8,9]. Furthermore, it was shown that microtubule stabilization is required for efficient DNA repair [10,11], revealing a link between microtubule dynamics and DNA damage response (DDR).

**Figure 1:**
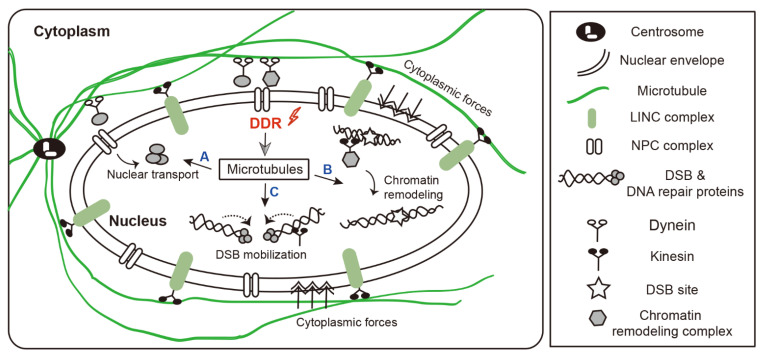
The role of microtubule dynamics in DNA damage response. Microtubules are involved in DNA damage repair at three main levels. (**A**) Centrosomes organize microtubules by controlling nucleation and anchoring processes. Cytoplasmic microtubules can mediate the transport of DNA repair factors into the nucleus. (**B**) Dynamic cytoplasmic microtubules physically interact with and exert mechanical forces onto nuclear envelope, which can impact nuclear morphology, leading to changes in chromatin structure. In addition, nuclear localization of microtubule components induces chromatin reorganization. These changes expose DNA damage sites, allowing access to various DNA repair proteins. (**C**) Microtubules/LINC complexes increase the mobility of damaged DNA and promote the recruitment of DNA repair proteins at the site of damage, which is important for DSB repair via both HR and NHEJ. Abbreviations: LINC, linker of the nucleoskeleton and cytoskeleton; NPC, nuclear pore complex.

Microtubules are involved in DNA damage repair at three main levels (Figure 1). First, microtubules are nucleated from the centrosome, which is usually located close to the nucleus in interphase cells. The filamentous network of microtubules extends throughout the cell and has an important role in multiple cellular processes. Cytoplasmic microtubules undergo post-translational modification that may alter their stability and function. These modifications appear to be especially important in the intracellular trafficking of DNA repair proteins. Second, cytoplasmic microtubules physically interact with and exert mechanical forces onto the nuclear envelope, which can impact nuclear shape, leading to changes in chromatin structure. In addition, nuclear localization of microtubule components induces chromatin remodeling. These changes expose DNA damage sites, allowing access to various DNA repair proteins. Lastly, the flexibility of the DNA double-strand break (DSB) end(s) is important for efficient DNA repair. Microtubules mobilize damaged DNA and promote interaction with repair proteins at the site of damage, which is important for DSB repair via both homologous recombination (HR) and non-homologous end joining (NHEJ).

In this review, we present the current state of knowledge in understanding the role of microtubule dynamics in DNA damage repair and discuss some perspectives gained by these discoveries towards genome stability.

## 2. Microtubule Organization and Stability in Response to DNA Damage

Microtubules are one of the three major cytoskeletal components in eukaryotic cells [2]. The centrosome, a major microtubule-organizing center (MTOC) in animal cells, comprises a pair of centrioles surrounded by pericentriolar material (PCM), which nucleates and anchors microtubules [1,3]. During mitosis, centrosomes form the spindle poles of the bipolar mitotic spindle, and during interphase, they nucleate the formation of the microtubule cytoskeleton [3,5,6]. The DDR proteins such as ataxia-telangectasia and Rad3 related (ATR), checkpoint kinase 1 and 2 (CHK1/CHK2), breast cancer susceptibility gene 1 and 2 (BRCA1/BRCA2), and RAD51 have been shown to localize in the nucleus and the centrosomes [12,13,14,15,16,17,18], implicating the existence of crosstalk between two organelles following DNA damage. Furthermore, recent studies demonstrated that microtubule stabilization is required for efficient DNA repair [10,11], revealing a link between the DDR and microtubule dynamics. In this section, we summarize the molecular mechanisms that regulate microtubule organization and stability in response to DNA damage during interphase. 

### 2.1. NEK2

Centrosome separation is critical for bipolar spindle formation and the accurate segregation of chromosomes during cell division [1]. NIMA-related kinase 2 (NEK2) is a centrosomal kinase required for accurate centrosome separation [19,20,21]. NEK2 activity fluctuates during the cell cycle, which is low in the G1 phase, peaking in the S and G2 phases [22]. The DDR pathway controlling centrosome separation is mechanistically linked to NEK2 [23]. IR-induced DNA damage results in the activation of ataxia-telangiectasia mutated (ATM) and phosphatase 1 (PP1). The increased activity of PP1 dephosphorylates and reduces NEK2 activity, leading to the inhibition of centrosome separation [24,25]. Thus, NEK2 might act as a downstream target of the DDR pathway that regulates centrosome separation and contributes to the G2 arrest under genotoxic stress [23].

### 2.2. Centrobin

Centrobin is a centriole-associated protein that is required for centriole duplication and elongation [26,27]. Centrobin also has a potential role in microtubule stabilization by interacting with α-tubulin [26,28,29,30]. Two different kinases have been shown to regulate the microtubule-stabilizing activity of centrobin [31,32]. Phosphorylation by PLK1 (polo-like kinase 1) stimulates centrobin to stabilize the microtubules during mitosis [31], while phosphorylation by NEK2 antagonizes the microtubule-stabilizing activity of centrobin during interphase [32]. As discussed earlier, NEK2 activity is decreased upon DNA damage [25], which therefore could increase microtubule-stabilizing activity of centrobin. Furthermore, centrobin was previously identified as a potential ATM/ATR substrate [33], suggesting a potential role of centrobin in DDR. A recent study has demonstrated that centrobin is phosphorylated in an ATR-dependent manner following ultraviolet (UV) exposure, and depletion of centrobin has a defect in UV-induced microtubule stabilization [34]. Within this context, it is proposed that ATM/ATR might be involved in regulating microtubule stability after DNA damage, at least in part, through centrobin and NEK2. However, further study is needed to identify the precise mechanism underlying the role of centrobin in DDR.

### 2.3. Pericentrin (PCNT)

PCNT is an integral centrosomal component that functions as a scaffold for anchoring numerous proteins in the centrosome [35,36]. Through this anchoring function, PCNT is involved in functional crosstalk between microtubule organization and DDR [37,38]. PCNT contributes to the microtubule organization in both interphase and mitosis. For instance, PCNT anchors the γ-tubulin ring complex (γ-TuRC) at spindle poles in mitotic cells, which is required for proper spindle assembly [39,40]. Loss of this anchoring mechanism induces a checkpoint response that prevents mitotic entry. PCNT is involved in DDR by mediating PCNT-dependent CHK1 localization at the interphase centrosome that regulates mitotic entry [37,38]. In addition, recent studies found that mutations in *PCNT* cause Seckel syndrome, defects in ATR-dependent DNA damage signaling, which displays mitotic failure and cell death [37,41,42,43], revealing PCNT functions in the DDR. Interestingly, it has been recently shown that chromatin remodeling proteins are involved in centrosome integrity [44]. Silibourne et al. identified chromodomain helicase DNA-binding protein 3 (CHD3), a component of the nucleosome remodeling deacetylase complex, as PCNT-interacting proteins and demonstrated that CHD3-PCNT complex is required for centrosomal localization of PCNT and for centrosome integrity [44].

### 2.4. CEP Family Proteins

Centrosomal protein of 63 kDa (CEP63) was first identified as a target of ATM/ATR following DNA damage [45]. ATM and ATR phosphorylate *Xenopus* CEP63 and promote its delocalization from the centrosome, inhibiting spindle assembly and delaying mitotic progression [45]. Furthermore, mutations of human *CEP63* were found in a primary microcephaly with defective ATR-dependent DNA damage signaling [46]. It has been also shown that CEP63 forms a complex with CEP152, another centrosomal protein implicated in microcephaly [46,47], and CEP63 deficiency leads to centriole loss due to impaired recruitment of CEP152 to the centrosome [48]. A centrosomal protein of 164 kDa (CEP164) is also phosphorylated by ATM/ATR upon DNA damage [49], which is required for DNA damage-induced CHK1 phosphorylation and G2/M checkpoints, indicating a critical role of CEP164 in ATM/ATR DNA damage signaling pathways.

### 2.5. αTAT1

The α-tubulin acetyltransferase 1 (αTAT1) catalyzes the acetylation of α-tubulin at lysine 40 (K40) in microtubules [50,51]. α-tubulin K40 acetylation has been shown to be enriched in stable microtubules, such as mitotic spindles and cilia [52,53]. A recent study demonstrated that α-tubulin K40 acetylation is induced following DNA damage and that αTAT1 catalytic activity is required for DNA damage checkpoint response [54], suggesting a potential role of α-tubulin K40 acetylation in DDR. Although the molecular mechanism by which αTAT1 affects DDR remains unclear, as α-tubulin K40 acetylation has been known to enhance the microtubule association of motor proteins and subsequent intracellular transport [55,56], αTAT1 may play a role in DDR, at least in part, by promoting nuclear transport of DNA repair proteins. HDAC6 (histone deacetylase 6) and SIRT2 (Sirtuin 2) are known to negatively regulate α-tubulin K40 acetylation [57,58]. Thus, it will be possible that reduced activity of HDAC6 and SIRT2 could contribute to the increase in α-tubulin K40 acetylation following DNA damage. However, it seems unlikely because it has been shown that deacetylating activity of HDAC6 or SIRT2 is required for efficient DNA repair [59,60], suggesting that αTAT1 is primarily responsible for inducing α-tubulin K40 acetylation in response to DNA damage.

## 3. Microtubule-Dependent Nuclear Remodeling Following DNA Damage

Recent studies in yeast and mammalian cells suggest that cytoplasmic actin and microtubules induce global changes in chromatin structure in response to DNA damage [61,62]. The microtubule dynamics are particularly important for chromatin segregation during mitosis; however, there is increasing evidence to suggest that microtubules are implicated in chromatin organization during interphase [7]. Recent studies have shown that, upon DNA damage, cytoplasmic microtubules can change the nuclear structure that provides a nuclear environment conducive to repair [8,63,64]. How can microtubule networks influence interphase chromatin upon DNA damage? Several links have been found between the microtubule network and the interphase chromatin. The first mechanism involves force transmission from cytoplasmic microtubules to chromatin through the nuclear envelope. The second mechanism involves nuclear accumulation of microtubule components that bind to chromatin and influence chromatin structure. The third mechanism involves microtubule-dependent nuclear transport of chromatin remodeling complexes. In this section, we highlight the role of microtubule dynamics in nuclear changes following DNA damage.

### 3.1. Microtubule-Driven Cytoplasmic Forces

The nuclear envelope is in close association with the microtubule networks. In interphase, centrosomes are usually located close to the outer surface of the nuclear envelope and interphase chromosome ends attach to the inner surface of the nuclear envelope [65,66]. Therefore, a physical link is expected to form between cytoplasmic microtubules and chromosomes. The nuclear envelope is connected to the different types of cytoskeletal elements by the linker of nucleoskeleton and cytoskeleton (LINC) complex formed by Sad1 and UNC84 (SUN) and Klarsicht/ANC-1/Syne homology (KASH) domain proteins [67,68,69]. SUN domain proteins span the inner nuclear membrane and interact with nucleoplasm and chromatin. KASH domain proteins are anchored in the outer nuclear membrane and interact with the cytoskeleton [67,68,69]. The interactions of SUN-KASH domain proteins across the nuclear envelope link the microtubule cytoskeleton to the nucleus [69]. The connection between cytoplasmic microtubules and the LINC complexes may allow the microtubule cytoskeleton to influence the nucleus by transmitting mechanical forces across the nuclear envelope. It has been shown that mechanical force applied to the nucleus induces direct stretching of chromatin, resulting in the activation of transcription [70]. Therefore, it is tempting to speculate that in response to DNA damage, microtubule tracks for intracellular transport may generate mechanical forces in the nuclear envelope, which may influence the nucleus and subsequently induce chromatin reorganization for DNA repair.

In eukaryotes, heterochromatin is generally located just beneath the nuclear envelope where it interacts with the nuclear lamina [71,72]. Nuclear lamina-associated heterochromatin has been shown to increase nuclear tension [73], which may provide nuclear stiffness [74]. As a consequence, microtubule-driven cytoplasmic forces to the nuclear envelope could be counteracted by the heterochromatin at the nuclear periphery. It is therefore speculated that balanced force along the nuclear envelope might play an important role in maintaining nuclear structure. Heterochromatin is markedly reorganized in response to DNA damage to control and facilitate DNA repair [75,76,77,78,79]. In particular, decondensation of heterochromatin has been observed in response to DSBs [76,78,80]. Among the mechanisms that may drive heterochromatin reorganization following DNA damage, ATM-dependent phosphorylation of the heterochromatin building factor KRAB-domain associated protein 1 (KAP1) results in dissociation of the chromatin remodeler CHD3, promoting chromatin relaxation [81,82]. This can result in moving away from a region containing heterochromatin, and possibly decreasing nuclear tension from the nuclear envelope-associated heterochromatin. Indeed, most recently, dos Santos et al. showed that DNA damage decreases nuclear tension through chromatin decondensation, which is required for genome stability [63]. Therefore, it would be possible that in the presence of DNA damage, nuclear morphology might be easily affected by the cytoplasmic forces, which in turn induces chromatin reorganization.

### 3.2. Microtubule and Microtubule-Associated Proteins

Recently, it has been shown that the chromatin remodeling complex has been implicated in microtubule organization [44,83,84,85,86,87]. Because chromatin-remodeling factors affect microtubule polymerization and spindle dynamics [44,85,87], it would be possible that microtubules and/or microtubule-associated proteins could be linked to chromatin reorganization.

#### 3.2.1. γ-Tubulin

In eukaryotes, there are five known tubulin isoforms, α-tubulin, β-tubulin, γ-tubulin, δ-tubulin, and ε-tubulin [88,89]. The α- and β-tubulin heterodimers assemble into dynamic microtubules and perform multiple important cellular functions. The γ-tubulin is essential for microtubule function, but it is not a component of microtubules. Rather, it is located at the centrosome and functions in the microtubule nucleation and microtubule polarity from the centrosome [1,3]. The δ-tubulin and ε-tubulin are required for triplet microtubule stability in centrioles and basal bodies [90]. Unlike α-tubulin and β-tubulin, γ-tubulin contains a nuclear localization sequence and a helix–loop–helix DNA-binding motif on the C-terminus [91]. There is growing evidence showing nuclear functions of γ-tubulin including transcription, chromatin remodeling, and DNA damage response. The genetic interaction between γ-Tub23C and SWItch/Sucrose Non-Fermentable (SWI/SNF) chromatin-remodeling complex has been shown in *Drosophila melanogaster* [92], suggesting a potential role of γ-tubulin in chromatin remodeling. In addition, nuclear γ-tubulin interacts with the transcription factor family E2 promoter-binding factor (E2F) and also with E2F DNA binding sites, leading to repression of E2F-induced transcription [93]. This can be similar to that of retinoblastoma protein (Rb), which exerts its tumor suppressor function primarily by inhibiting the E2F transcription factors [94]. Interestingly, besides interacting with E2Fs, Rb inhibits E2F-induced transcription by recruiting chromatin remodeling factors (histone deacetylases and SWI/SNF complexes) and DNA methyltransferase DNMT1 [95,96,97]. It is thus tempting to speculate that γ-tubulin may be involved in the recruitment of DNA-remodeling factors in the nucleus. Furthermore, γ-tubulin interacts with DNA repair protein RAD51 and forms a nuclear complex with BRCA1 after DNA damage [98], suggesting a link between DNA repair and the microtubule networks.

#### 3.2.2. KIF4

A major group of molecular motors involved in intracellular transport are kinesins named KIF (kinesin superfamily protein). There are several dozen KIFs in mammalian cells to constitute at least 14 kinesin families [99,100]. The majority comprises two domains: an ATP hydrolysis domain that allows it to traverse microtubules, and a tail domain that is able to bind to structures and/or cargos [99,100]. KIF4 is a microtubule-bound motor protein that associates with chromosomes and microtubules during mitosis and contributes to faithful chromosome segregation [101]. However, KIF4 is unique among kinesins in that it localizes to the nucleus throughout interphase [102,103], suggesting its non-mitotic function. It has been shown that KIF4 is important for chromatin organization, transcription, and DNA repair. There are several mechanisms for the nuclear functions of KIF4. (1) KIF4 induces chromatin condensation by inhibiting Poly [ADP-ribose] polymerase 1 (PARP1) activity, which maintains an open chromatin architecture through Poly ADP-ribosylation (PARylation) [101,104,105]. (2) KIF4 promotes nucleosome assembly by recruiting histone chaperones and chromatin remodeling complexes to newly synthesized DNA, leading to chromatin compaction [103]. (3) KIF4 negatively regulates transcription by interacting with transcriptional repressive complexes such as DNMT3B and HDAC1 [103,106]. (4) KIF4 is involved in HR repair by interacting with BRCA2, which promotes the recruitment of RAD51 to DSBs sites [107,108]. Taken together, KIF4 performs nuclear functions that can change chromatin structure during interphase, in addition to its known function as a microtubule-bound motor protein during mitosis.

#### 3.2.3. Actin

Actin, one of the cytoskeletal proteins, is also present in the nucleus and is associated with soluble nuclear proteins [109,110,111,112]. It has been shown that actin and actin-related proteins are integral components of several chromatin remodeling complexes, such as SWI/SNF complexes and the INO80-containing complexes [110,111]. Although the roles of actin and actin-related proteins in the complexes are yet unclear, it is speculated that they play crucial roles in maintaining the integrity of the protein complexes in the chromatin and thereby affecting chromatin structure and accessibility.

## 4. Microtubule-Dependent Chromatin Mobility Following DNA Damage

Recent studies in yeast suggest that DSB repair is thought to involve the broken ends being moved to ‘repair centers’ in the nucleus and indicate that DDR-dependent chromatin mobility promotes HR repair [113,114,115,116]. However, the mechanism by which the DNA damage promotes increased chromatin mobility remains to be elucidated. One clue might be found in the recent observation that DNA damage-dependent phosphorylation of nucleoporins releases the interaction between tethered chromosomes and the pore [117]. Another possible mechanism could involve the chromatin remodeling complex. DSB recruitment of chromatin remodeling factors such as INO80 may be important to promote the increase in chromatin mobility [118]. Whatever the mechanism, DNA damage-induced chromatin mobility is required to increase DNA repair efficiency. However, it should also be noted that DSB mobility is not always positive, because increased mobility might lead to unwanted translocations between chromosomes, increasing genome instability. In this section, we discuss the emerging role of microtubule dynamics in chromatin mobility near the DSB sites.

### 4.1. Chromatin Mobility during HR and NHEJ

Double-strand breaks can be repaired via either HR, the exchange of genetic material between homologous DNA sequences, or NHEJ, the direct ligation of the broken DNA ends [62,119]. HR is an error-free pathway that predominantly occurs in the late S and G2 phases, whereas NHEJ is an error-prone repair pathway that can occur throughout all cell cycle phases [62,120]. The bacterial genomes experience constant pressure from multiple DNA damaging stresses. The bacterial response to DNA damage is known as the SOS response [121,122]. There are two main proteins involved—one, LexA, to keep the response switched off while the cell is healthy, and the other, RecA, to turn it on when DNA damage occurs [123]. In bacteria, DSB repair mostly relies on HR, which involves the action of bacterial recombinase protein RecA [121,122,124]. Interestingly, a number of bacteria have evolved additional DNA protection mechanisms provided by small bacterial DNA-binding proteins, namely, nucleoid-associated proteins (NAPs; e.g., HU, DNA-binding protein from starved cells (Dps)), small acid-soluble spore proteins (SAAPs), and single-stranded binding proteins (SSBs) [125,126,127,128,129,130,131,132,133,134]. These small DNA-binding proteins are able to protect bacterial genomic DNA by the formation of nonspecific protein–DNA complexes, which could be linked with efficient DNA damage repair. Together, both DNA damage repair and DNA protective binding ensure genome stability in bacteria. Yeasts preferentially use HR [62]. Increased chromatin mobility in response to DNA breaks has been reported in yeast [113,114,115,116]. The mobility of damaged chromatin depends on the Mec1ATR kinase, resection of the DSB ends, and the RAD51 recombinase [135,136]. Increased ability of RAD51-DSB ends to find a homologous sequence promotes an efficient HR repair [113,116,137]. As discussed in the previous section, DNA-damage-induced chromatin reorganization can promote the extrusion of DSB sites from the heterochromatic domain and increase access to repair factors. As a consequence, DSB ends become more mobile, which can facilitate homology search and repair. A recent study in *C. elegance* has shown that LINC complexes facilitate DSB repair through both the inhibition of NHEJ and the promotion of HR [138]. Based on the data, Lawrence et al. have proposed a model whereby the LINC complex can both directly inhibit the KU70/KU80/DNA dependent protein kinase (DNA-PK) complex through SUN proteins and license HR repair through microtubules [138].

NHEJ that relies on the direct rejoining of broken ends is more predominant in mammalian cells [62]. Compared to yeast, DSBs are thought to be immobile within the mammalian cell nucleus [139,140,141]. However, deprotected telomere ends have increased mobility compared with protected telomeres [142]. This increased mobility depends on both ATM and p53-binding protein 1 (53BP1), and these ends are repaired through NHEJ. Recently, Lottersberger et al. revealed that uncapped telomeres and irradiation-induced DSBs in mouse cells exhibit increased DSB mobility that is dependent on dynamic microtubules, 53BP1, the LINC complex, and the motor protein kinesins [143]. In this study, Lottersberger et al. have proposed a model whereby dynamic cytoplasmic microtubules with the LINC complexes can “poke” the nucleus and increase the mobility of 53BP1-associated damaged DNA for NHEJ repair [143]. A recent investigation employing a single-particle tracking method in yeast further demonstrated the requirement of microtubules in DSB mobility upon DNA damage [144].

### 4.2. KIF2C

As described in the previous section, there are different kinesin proteins localized inside the nucleus, although their roles are largely unknown. Cytoplasmic microtubules can affect the DDR either by the nuclear transport of repair factors [11] or by association with the LINC complexes [143], whereas nuclear kinesins are likely to be involved in a more direct manner. Kinesin family member 2C (KIF2C), also known as mitotic centromere-associated kinesin, is a microtubule-dependent motor protein with a variety of important cellular regulatory functions, such as the regulation of mitosis and genome stability [145,146,147]. Interestingly, it has been recently demonstrated that KIF2C is required for efficient DSB repair via both HR and NHEJ [148]. Depending on its microtubule depolymerizing activity, KIF2C is recruited to DSB sites, promoting DSB mobility and forming DNA damage foci in an ATM-dependent manner, suggesting that KIF2C serves as an important DDR factor that mediates the local mobility and dynamics of DSB ends.

### 4.3. KIF18B

Kinesin family member 18B (KIF18B) is another member of kinesin that localizes to the nucleus and binds to chromatin throughout the interphase [149]. Most recently, KIF18B, which is also recruited to the sites of DSBs, was reported to be required for 53BP1-mediated DSB repair [150]. This study demonstrated that the ability of KIF18B to bind 53BP1, as well as its motor function is required for efficient 53BP1-mediated end-joining of DSBs.

### 4.4. DNA-PK-AKT

Ma et al. have recently reported the effect of DNA damage on microtubule dynamics. The authors discovered that DSBs promote microtubule dynamics in G1 cells through DSB-induced microtubule dynamics stress response, which occurs in a DNA-PK-AKT-dependent manner [151]. As a consequence, increased microtubule dynamics promote DSB mobility and facilitates NHEJ repair in G1 cells.

## 5. Conclusions

**Figure 2:**
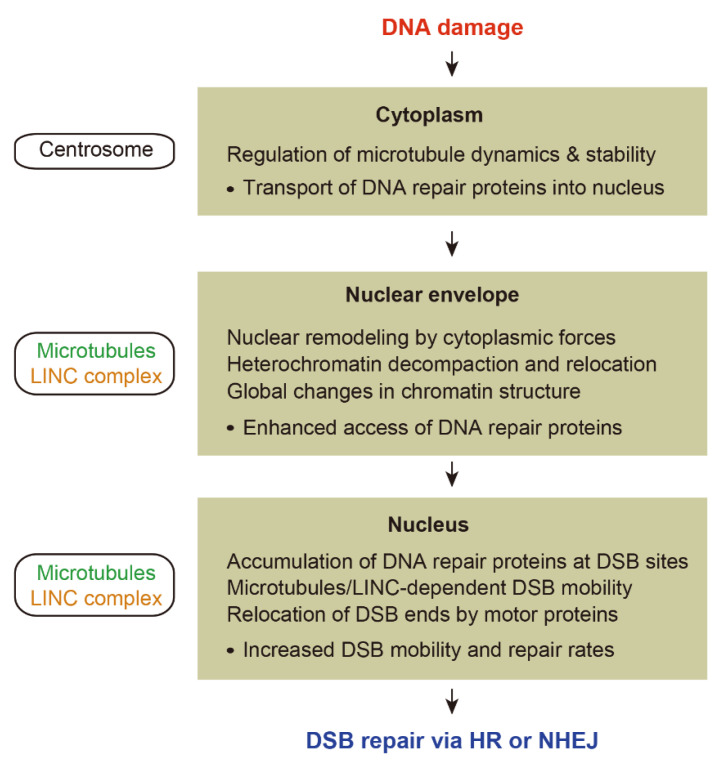
Overview of the role of microtubule dynamics in DNA damage response.

This review highlights recent studies investigating the role of microtubule dynamics in DNA damage repair with a focus on the connections between DDR and microtubule networks (Figure 2).

We first focused on proteins directly related to microtubule organization and stability in response to DNA damage, in which centrosomes play a central role in the regulation of microtubule organization as part of the DDR. We then discussed how cytoplasmic microtubules are involved in the nuclear reorganization following DNA damage. Microtubules can impact nuclear architecture either by generating the microtubule-driven mechanical forces or facilitating the nuclear import of proteins involved in chromatin reorganization. Lastly, we highlighted the molecular mechanisms underlying microtubule-dependent chromatin mobility during DNA repair. Increased mobility of damaged chromatin appears to play an important role for both HR and NHEJ.

Microtubule-targeted agents (MTAs), such as paclitaxel and vinblastine, can induce mitotic catastrophe in cancer cells by disrupting the mitotic spindle [152,153]. Interestingly, MTAs display greater efficacy than mitosis-specific inhibitors, suggesting that MTAs can inhibit both mitotic and non-mitotic functions of microtubules [152,153,154]. MTAs are very effective in cancer treatment when used in combination with DNA-damaging agents [11]. This may be attributed to the ability of MTAs to interfere with microtubule-dependent DNA damage repair. In addition to their antimitotic effects, MTAs can elicit an immune response following the disruption of microtubules [155]. As many cancer cells often overexpress immune checkpoint proteins, thus escaping cancer immune surveillance, immune checkpoint inhibitor (ICI)-based therapy is designed to strengthen cancer immune surveillance [156,157]. As discussed earlier, MTA treatment can stimulate immune responses [155]; thus, combining with ICIs could enhance the antitumor activity of MTAs by stimulating immune surveillance. Indeed, several clinical trials are ongoing to test the combining effect of taxanes and ICIs [158,159,160].

Given their central role in the therapy of cancer, MTAs will continue to be used widely in combination with other anticancer drugs. Thus, there is a critical need to identify novel tubulin-targeting drugs with improved properties that can be used as anticancer agents. In addition, it will be important to select cancer groups that can receive the maximum benefits of a combination with MTAs for cancer therapy, which may provide tumor selectivity.
